# Red Blood Cells’ Area Deformation as the Origin of the Photoplethysmography Signal

**DOI:** 10.3390/s23239515

**Published:** 2023-11-30

**Authors:** Lucian Evdochim, Eugen Chiriac, Marioara Avram, Lidia Dobrescu, Dragoș Dobrescu, Silviu Stanciu, Stela Halichidis

**Affiliations:** 1 Department of Electronic Devices, Circuits, and Architectures, Faculty of Electronics, Telecommunications and Information Technology, University Politehnica of Bucharest, 060042 Bucharest, Romania; lidia.dobrescu@upb.ro (L.D.); dragos.dobrescu@upb.ro (D.D.); 2 National Institute for Research and Development in Microtechnologies—IMT Bucharest, 077190 Voluntari, Romania; eugen.chiriac@imt.ro (E.C.); marioara.avram@imt.ro (M.A.); 3 Laboratory of Cardiovascular Noninvasive Investigations, Dr. Carol Davila Central Military Emergency University Hospital, 010242 Bucharest, Romania; silviu.stanciu@umfcd.ro; 4 Department of Clinical Medical Disciplines, Faculty of Medicine, Ovidius University of Constanta, 900527 Constanta, Romania; stela.halichidis@univ-ovidius.ro

**Keywords:** photoplethysmography origin, red blood cell shape deformation, mathematical transfer function, microcirculation, vasomotor activity

## Abstract

The origin of the photoplethysmography (PPG) signal is a debatable topic, despite plausible models being addressed. One concern revolves around the correlation between the mechanical waveform’s pulsatile nature and the associated biomechanism. The interface between these domains requires a clear mathematical or physical model that can explain physiological behavior. Describing the correct origin of the recorded optical waveform not only benefits the development of the next generation of biosensors but also defines novel health markers. In this study, the assumption of a pulsatile nature is based on the mechanism of blood microcirculation. At this level, two interconnected phenomena occur: variation in blood flow velocity through the capillary network and red blood cell (RBC) shape deformation. The latter effect was qualitatively investigated in synthetic capillaries to assess the experimental data needed for PPG model development. Erythrocytes passed through 10 µm and 6 µm microchannel widths with imposed velocities between 50 µm/s and 2000 µm/s, according to real scenarios. As a result, the length and area deformation of RBCs followed a logarithmic law function of the achieved traveling speeds. Applying radiometric expertise on top, mechanical-optical insights are obtained regarding PPG’s pulsatile nature. The mathematical equations derived from experimental data correlate microcirculation physiologic with waveform behavior at a high confidence level. The transfer function between the biomechanics and the optical signal is primarily influenced by the vasomotor state, capillary network orientation, concentration, and deformation performance of erythrocytes.

## 1. Introduction

The photoplethysmography (PPG) technique is a century-old technique, first mentioned by the German scientist M. R. Bonsmann as the photocell method in 1934 [1] for pharmacological studies. Alrick Hertzman further investigated this novel tool to correlate the obtained waveform with physiological behavior [2]. His studies involved both reflective and transmissive modes, aiming to develop a physiological model to support the origin of the recorded optical signal. However, technology limitations and the state of the art of blood circulation knowledge slowed down further investigation at that time. Therefore, the source of the signal was linked to blood volume variation at the microcirculation level as vasomotor activity was well correlated with the recorded waveform baseline. Another additional assumption arose from skin micropulsation being induced by large vessels’ wall movements with every cardiac cycle. Based on experimental observations, he tried to separate the active (AC part) and passive components (DC part) of the waveform to correlate with the arterial and venous blood flow [3]. The emerged conclusion points out that the amplitude of the PPG signal is a direct measure of arterial tone, maintaining his belief that the amount of blood supply within the analyzed tissue is directly proportional to the absorbed light flux. This led to a simplistic model, popular at that time, which allowed Takuo Aoyagi to develop pulse oximetry in the 1970s [4]. This device was intended to measure blood oxygen saturation levels (SpO2), an important physiological marker for anesthesia management. Ten years later, it was introduced into the US hospital environment as a fingertip sensor, becoming a commercially available device for non-clinical use a few years later.

Despite the proven feature obtained from the PPG technique, the medical community wondered if the recorded signal could extract more cardiovascular or hemodynamic insight intended for better patient care. The unfiltered signal is very complex, being modulated by physiology systems like the cardiac, respiratory, and autonomic systems. Thus, the absolute numbers of waveform amplitude cannot be compared between two patients without a defined calibration protocol. In the field of anesthesia [5,6,7], many questions arose regarding PPG waveform interpretation for clinical applications. Even though new knowledge was generated about blood circulation mechanisms during that time, no significant updates regarding photoplethysmography origin have been made.

The development of novel noninvasive hemodynamic tools, such as videocapillaroscopy [8,9], has provided a clear image of the microcirculation process. Multiple pieces of evidence show that capillaries have a fixed volume capacity without any sign of elastic proprieties compared to arteries or arterioles. Since the blood supply that branches to the peripheral network inherits a pulsatile manner modulated by the cardiac cycle, according to the flow conservation theory, the velocity parameter follows the same trend too. On top of this, the flow distribution through different capillary clusters is intermittently modulated by the precapillary sphincters [10,11,12]. This biological mechanism is intended to supply different tissue sites when there is a demand for nutrients. In this way, the arterial network achieves optimization compared to the scenario of equally sending blood flow to all capillaries by neglecting nutrient saturation states. In the human body, plasma travels through these tiny vessels within a velocity range of 50 µm/s to to 2 mm/s. The final value within this spectrum is highly influenced by physiological feedback mechanisms like vasoconstriction and vasodilation [13,14,15,16]. According to the conservation flow theory, the resulting pulsatile part, due to the cardiac cycle, has a peak-to-peak value from 20 µm/s up to 500 µm/s. This continuous variable flow process, achieved by changing both baseline and amplitude values, is designed to maintain a proper capillary pressure during the gas exchange process.

**Figure 1:**
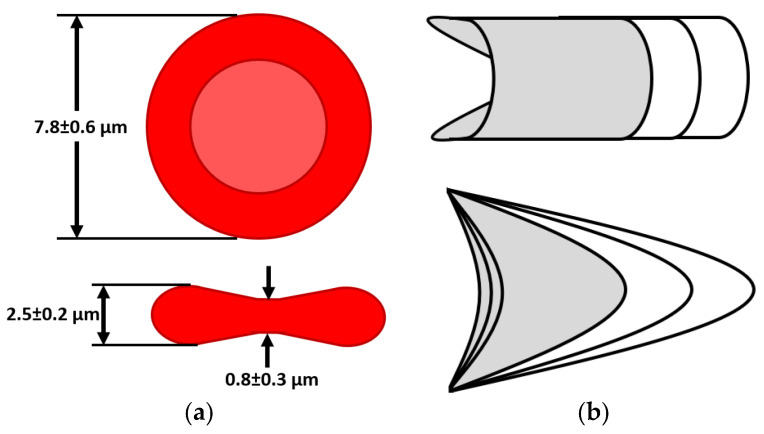
(**a**) The usual RBC size in healthy individuals in the steady state. (**b**) Modes of RBC deformation proportional to flow velocities and available space: top—*bullet* shape; bottom—*paraboloid* shape.

Another microcirculation process involves the traveling mode of red blood cells (RBCs) within the plasma flow. These microelements, being one of the most important constituents of the blood, exhibit interesting properties compared to the central circulation regime. Firstly, erythrocytes travel through capillaries as a group, a mode called *roleux* [17,18], generating gaps within the plasma. Computational simulations [19,20] indicate that this flowing mode decreases the shear stress sensed by the moving elements. Secondly, the cells have the ability to deform their geometry proportionally to the flow gradient. During the resting state or at lower speeds, RBCs maintain a *diskette* shape, as shown in Figure 1a. On the other hand, at high velocities, erythrocytes begin to deform along the longitudinal axis, turning into *bullet* or *paraboloid* shapes, as depicted in Figure 1b [21,22,23,24]. This behavior has been demonstrated in both in vivo and in vitro studies, reaching another level of knowledge maturity. The elastic property is also well conserved during the blood storage process for blood intended for transfusion [25,26]. On top of this, the erythrocytes’ deformation into another stable shape imposed by velocity stimuli can occur in under 1.5 ms.

The study of the interface between mechanical peripheral circulation and the optical nature of PPG is supported by various experimental observations. Since the dermis layer contains not only nail-fold capillary loops but also other biological elements, it forms a nonhomogeneous layer. Thus, any type of external energy, including light from the visible spectrum, undergoes a certain amount of attenuation. The popular green mode used in pulse oximeter sensors barely passes the epidermis, reaching a depth of only 0.5 mm into the skin [27,28]. On the other end of the spectrum, near-infrared light (NIR) performs better by reaching up to 2 mm [29,30]; this is known as the biological window. Thus, it achieves a sufficient path length for illuminating the entire capillary height of about 500 µm. In the context of the photoplethysmography technique, this dermis area level is the main source for extracting optical information.

With these newer mechanical and optical insights regarding microcirculation, no commonly agreed PPG model has been developed to link the origin of the pulsatile signal and physiological mechanism within the peripheral site. The initial model linked to blood volume variation is valid for the recorded waveform baseline as precapillary sphincters control the supply to tissue zones. However, it cannot explain the pulsatile nature of the recorded signal. Paradoxically, the pulse oximeter signal is inverted on the y-axis with respect to flow velocity, which reaches maximum values at the systolic (SYS) phase and minimum in the diastolic (DIA) phase.

Alternative PPG models propose RBC orientation during blood flow [31], tissue compression due to arteriole wall pulsation [32,33], or diffuse absorption by the tissue layer in combination with specimen concentration [34,35]. Even if these proposals result in a plausible explanation for the pulsatile nature, they do not provide a mathematical or physical equation. In fact, the major issue between mechanical and optical interfaces is represented by the defining of a suitable transfer function. In other words, PPG is just an interrogation method of biomechanics inside the circulation network. In the absence of an eligible model, not only are predictions of optical waveform behavior limited but, also, the interpretation of the recorded signal’s morphology is limited. As shown in a few studies [36,37], PPG is greatly affected by reduced blood oxygen saturation levels and by increased arterial blood pressure (ABP) values. Thus, an important benefit of defining a suitable model would be to improve the clinical use of the aforementioned technique, especially in the domain of wearable devices intended for improved personalized healthcare.

The presented study aimed to investigate PPG waveform origin, setting the starting point from the discussed microcirculation property: variable flow velocity through capillaries concomitant with RBC shape deformation. In the first part of the results, experimental qualitative data were obtained by in vitro investigation while, in the second one, a transfer function was developed by considering all the known physiological findings. The scope of the current study is to link hemodynamic behavior to the resulting optical signal recorded from light interaction.

## 2. Materials and Methods

**Figure 2:**
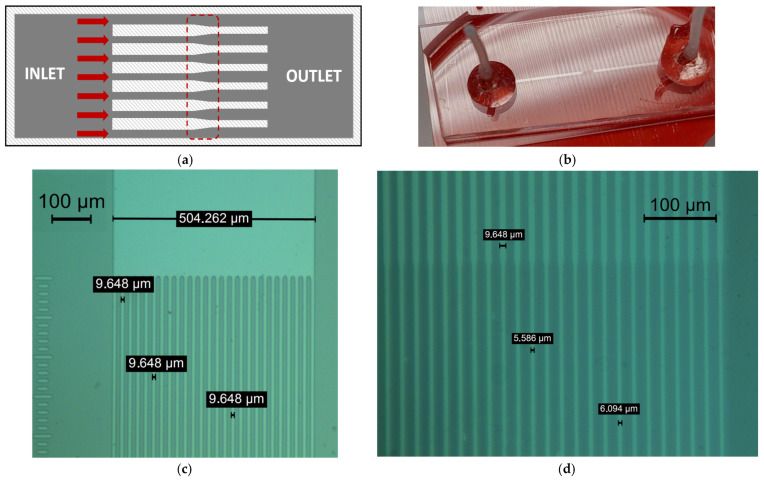
(**a**) Design concept of the synthetic capillary network; with the red arrows, the direction of imposed flow is shown within 10–6 µm channel widths; with the red dotted line, the transition region is highlighted. (**b**) Resulting synthetic capillary network (translucent mid-channel) with attached ports. (**c**) Resulting 10 µm channel width after the fabrication process; magnification 10× factor. (**d**) Resulting 6 µm channel width after the fabrication process; magnification 15× factor.

The initial phase in unraveling PPG origin involves an experimental examination of red blood cell deformation within a synthetic capillary network. The constructed vessels were specifically designed to mimic human pathology, with a particular emphasis on two widths: 10 µm and 6 µm. A transitional zone was incorporated in between, illustrated in Figure 2a, where each section spans a length of 500 µm. This dimension closely approximated the extent of an unfolded capillary loop. The fabrication of the capillary array employed the soft lithography process, utilizing polydimethylsiloxane (PDMS) material. Constructing the microchannel mold started with applying a positive photoresist on a silicon wafer, followed by exposing the photolithographic mask under UV light. Next, the wafer was etched by using deep reactive ion etching (DRIE) with a Bosch process. To render the silicon mold hydrophobic, 1 mL of chlorotrimethylsilane was applied in a closed environment. The PDMS base and curing agent were then prepared in a 10:1 ratio. The mixture was degassed and poured on the microchannel mold following heating in the oven at 90 °C for an hour [38,39]. Following this, PDMS was carefully peeled off the mold and drilled at the designated locations for the microfluidic ports. A reactive ion etching (RIE) process involving O_2_ plasma treatment at a low power of 20 W for 20 s was employed before sealing the material with a glass slide. Finally, microfluidic ports were attached using epoxy, as illustrated in Figure 2b. Due to the inherent variations in the fabrication process, the resulting synthetic capillaries exhibited average widths of 9.5 µm and 5.6 µm, respectively, as depicted in Figure 2c,d.

Erythrocytes were extracted from blood samples obtained from healthy volunteers and suspended in a saline solution with a concentration of 0.9% to preserve cell membrane integrity. The flow rate of the resulting mixture through the capillary network was precisely controlled using the electronic pressure pump Mitos 400 P. Using a microscope with a magnification level of 20× and digital storage capability for recorded frames, 8 MP resolution at 30 FPS, an initial calibration procedure was initiated for pressure-flow rate function. The objective was to impose RBC velocity values between 50 µm/s and 2 mm/s, which aligns with the typical circulation dynamics in the human body.

**Figure 3:**
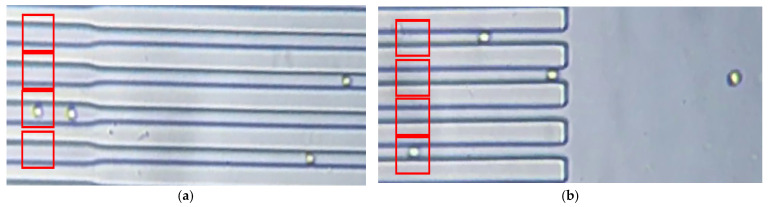
(**a**) Video frame of the recorded RBC deformation through the synthetic capillary network. The transition zone is also visible. With the red rectangles, ROI were placed across the individual channel. (**b**) The interface between the 6 µm channel and the free zone space (outlet side).

The processing and analysis of the video frame data were carried out using the Matlab 2023a software edition. Instances of cell movement were recorded in both capillary widths, as shown in Figure 3a, and in the free zones at the interfaces with microchannels, as shown in Figure 3b. Measurements of the erythrocytes’ length and area deformation were conducted in predefined regions of interest (ROI). For every cropped ROI, the edge detection method was employed to distinguish cells from channels using the Matlab *edge* function. The obtained cells’ contours were dilated inwards with a custom algorithm to fill the erythrocyte area accordingly. At this point, the RBC became a discriminant element that was easily tracked in each video frame. The mapping factor between the physical dimensions and the recorded pixels was based on the following principle: the known width of each channel is represented by a finite number of vertically aligned pixels, as depicted in Figure 3. Thus, with this correspondence, the mapping factor was computed to translate pixel measurements into physical quantities. In this way, the estimations of RBC length and area during their travels through the capillary network were recorded.

## 3. Results

### 3.1. Deformation Experimental Data

The experiment’s outcomes are presented in the form of a length and the corresponding area deformation function relative to the traveling speed through both types of capillaries. However, due to erythrocyte clots or adhesion to the PDSM wall material, some microchannels became clogged, making the corresponding ROI inactive. This undesired effect increased the initial baseline velocity value as the flow rate was redistributed to the remaining available paths. The investigation ran on several sets of channel networks to collect sufficient data, as aimed for in the previous section.

**Figure 4:**
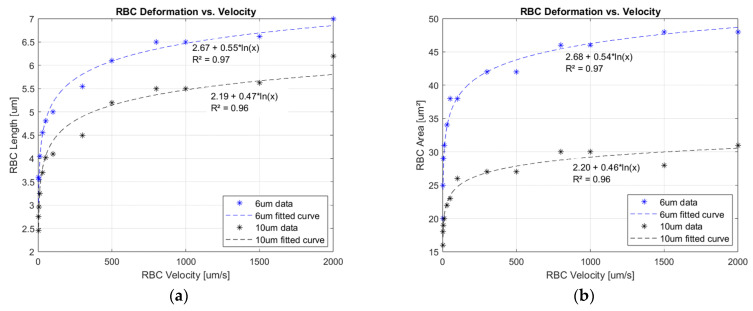
(**a**) RBC average length deformation against measured flow velocities. (**b**) Average area deformation against measured flow velocities.

In Figure 4a, the first parameter of interest, RBC average length, is plotted against the measured flow velocities. A significant observation is that the elongation adheres to a logarithmic law multiplied by a deformation factor, as illustrated by the interpolation function. At zero velocity, the longitudinal length of the cell is, on average, 2.5 µm, as previously shown in Figure 1a. Consequently, this parameter is manifested as a positive offset cumulated with the logarithmic trend. The same physiological behavior also occurs in RBC lateral area deformation, as shown in Figure 4b. Since the cell conserves total mass, achieving positive area deformation implies a reduction in another dimension. According to Hook’s law, material elongation corresponds to a decrease in thickness. Thus, in the experimental set-up, RBCs shall reduce the wall thickness to attain a paraboloid shape proportional to the imposed velocity. In simpler terms, the cell increases the hollow volume inside the chosen shape.

Another important finding is represented by the deformation saturation of the erythrocytes. Further increases in flow velocity will weakly influence the change in any dimension. In the case of the 10 µm channel, after a length modulation of 5 µm, cell deformation is slowed, as depicted in Figure 4a. In the 6 µm channel, constrained to deform on the width axis, the threshold point occurs at around 6 µm and continues to increase slowly up to 7 µm. It was expected that the RBC would not deform indefinitely due to its internal mechanical structure, having a limited deformation reserve. It is important to highlight that the deformation effect implies an increasing plasma gap between the cell’s membrane and channel wall, as demonstrated in other similar investigations [21,22].

Next, summarizing the experimental data, the mathematical law involved in the deformation effect of the red blood cells follows the form:

(1) $$D=D_{0}+aln\left(v\right),$$

where D is the final observed dimension, length, or area; D_0_ represents the dimension of interest at zero flow; and *v* is the velocity of the cell. Factor *a* represents the deformability modulus, indicating the degree to which RBCs can easily or resistively deform into their final shape. Studies conducted on the transfusion topic [25,26] observed a progressive degradation of the deformability index (DI) with erythrocyte age, measured in terms of blood sample storage time. Moreover, a reduced DI was observed in patients with diabetes mellitus (DM) and coronary artery disease [40,41,42]. This impaired rheological property was linked to an increased risk of cardiovascular disorders (CVDs). In the context of the current study, a lower *a* factor will prevent RBCs from changing from a *diskette* to a *bullet* shape. As an effect, the zones with capillary diameters in transition, especially those under 6 µm, would be predisposed to clotting. This defined mathematical equation, Equation (1), proposes the foundation of the PPG origin.

### 3.2. The Photoplethysmography Mathematical Model

The development of the transfer function between the observed experimental data and the recorded optical PPG signal utilized physical quantities from the radiometry domain [43,44]. For this step, two main assumptions are defined regarding the involved biological specimens:

- The dermis layer is treated as a diffuse reflector. Therefore, the received radiant power is just scattered. This approximation is provided by the collagen fiber network, which possesses this property [45];
- Only red blood cell elements suspended into the capillary nailfold geometry possess absorptance properties [46,47]. No other biological elements, such as glands, lymphatic channels, and nerves, are considered.

**Figure 5:**
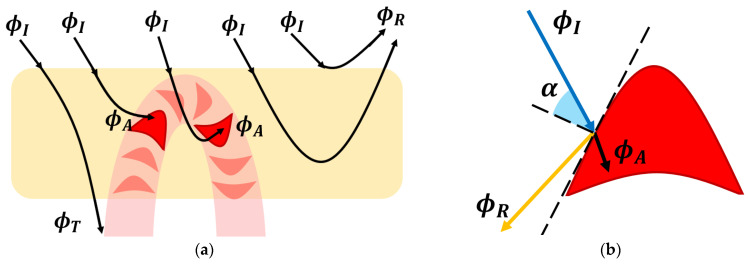
(**a**) Interaction between the incident light beam and the biological specimens: tissue and red blood cell. Multiple event possibilities are presented from left to right. (**b**) Qualitative interaction between the light beam and the erythrocyte element.

The Beer–Lambert equation is not suitable for the chosen model since the above-imposed approximations do not fulfill the law conditions. The aim is to understand waveform appearance by analyzing the interaction between incident light flux and RBC behavior over time. Hence, the starting point begins with the conservation law of the light flux power:

(2) $$\phi_{I}=\phi_{R}+\phi_{A}+\phi_{T},$$

where $\Phi_{I}$ is the incident light flux power that comes from the pulse oximeter sensor and enters into the dermis layer. The recorder beam $\Phi_{R}$ is the reflected flux, which could have multiple paths, as depicted in Figure 5a. The amount of radiant power absorbed by a single erythrocyte is labeled with $\Phi_{A}$. The light flux that moves away from the sensor location on the opposite plane is labeled with $\Phi_{T}$ as the transmitted flux. In the reflective mode of the PPG device, this light beam is lost information about interactions with biological layers. Therefore, in the next developed mathematical equations, this factor will be discarded since, for the presented context, it cannot be recorded.

Every solid element, even a deformed RBC, will absorb the radiant power to some degree, only with the exposed surface pointed to the incident light beam. According to the Poynting vector theory in the radiometry context, the amount of received radiation by the illuminated object is proportionate to the incident flux multiplied by the cosine angle between the beam and surface normal, as shown in Figure 5b.

Since the erythrocyte surface follows a parabolic trend, a straight light beam will encounter various angles depending on the irradiance location. Therefore, the total amount of absorbed radiant power will be equal to the area projection on the tangent line, labeled with A, and the average value of the cosine angle α, as follows:

(3) $$\phi_{A}=\phi_{I}\gamma_{\lambda }A\cos(\alpha ),$$

where $\gamma_{\lambda }$ represents the effectiveness of the cell absorption property of the incident radiant energy by a given energy wavelength λ. Since the light sources used in the pulse oximeter are of the incoherent type, every photon frequency will contribute by a different amount. The interaction of hemoglobin protein with the visible light spectrum is also known under different blood oxygen saturation ratios [48,49]. Due to the larger illuminated dermis volume in relation to the cell’s physical size, a cluster of capillaries is irradiated by the pulse oximeter sensor. So, multiple cells are targeted where each one contributes to the total absorption quantity by shape orientation with respect to the incident light flux direction. The total number of irradiated erythrocytes by the pulse oximeter sensor is annotated with the *N* parameter. By replacing Equation (3) with Equation (2), where the transmitted flux is discarded, the amount of the remaining reflected quantity will be:

(4) $$\phi_{R}=\phi_{I}\left[1-\sum_{i=1}^{N}\gamma_{\lambda }A_{i}\cos\left(\alpha_{i}\right)\right]\;$$

From the resulting equation, it is noticed that the number of irradiated erythrocytes will modulate the baseline of the received PPG signal, specifically the DC part. At this point, RBCs under analysis are considered to be motionless. The amount of plasma mass cumulated into the fixed volume of the capillary network is always constant during one cardiac cycle, in the absence of the vasomotor mechanism. This effect allows changes in the hemoglobin concentration within a fixed delimited volume. Usually, the rapid upstroke of the blood flow during the systolic phase leads to temporal RBC clustering. Few pulse-oximeter-related studies observed decreased accuracy in anemic patients where the concentration of hemoglobin cells per volume unit was smaller compared to normal patients [50,51]. Developing further the mathematical equation by adding the experimental observation law, Equation (1) regarding deformability, the following relation is obtained:

(5) $$\phi_{R}=\phi_{I}\left[1-\sum_{i=1}^{N}\gamma_{\lambda }\left(A_{0;i}+a_{i}\ln\left(v_{i}\right)\right)\cos\left(\alpha_{i}\right)\right]\;$$

The total amount of received light flux (5), considering biomechanics, is inversely proportional to the deformation degree of the illuminated cells. In the stationary case, where RBC velocity *v* is equal to zero, the logarithmic member is canceled, leading to the previous Equation (4) description. Thus, the baseline value of the optical signal, the DC part, is just affected by the erythrocytes’ presence. On the other side, at flow state, recorded light flux begins to be modulated in time by the logarithmic member; the velocity term is variable during each cardiac cycle, according to the imposed blood flow during the SYS and DIA phases. This variation of the parameter builds the alternative component, the AC part, of the PPG signal. At this point, it can be noticed that the maximum value of RBC velocity, reached during the SYS phase, results in a minimum reflected light flux $\phi_{R}$. During the DIA phase, the opposite effect occurs, resulting in the inverted nature of the recorded PPG signal with respect to the cardiac cycles. In order to highlight the DC and AC parts as standalone components, the above equation is split and approximated to:

(6) $$\phi_{R}\left(t\right)=\left\{\begin{array}{c} \approx -\gamma_{\lambda }NA_{0}\cos\left(\alpha \right),\;\;v=0\\ \approx -\gamma_{\lambda }Naln\left(v\right)cos\left(\alpha \right),\;\;v>0 \end{array}\right.$$

**Figure 6:**
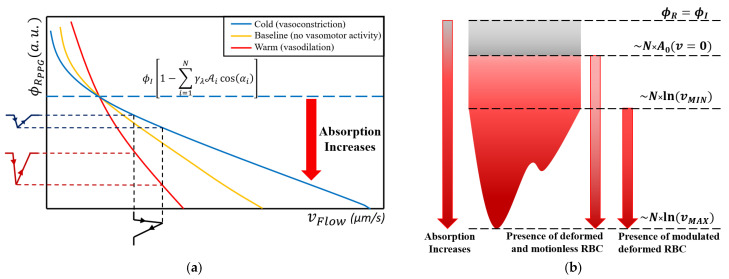
(**a**) Transfer function between mechanical flow rate inside the capillary network and the recorded PPG optical signal. The upper side of the dotted blue line delimits the signal validity boundary. Above this limit, the signal does not exist: reflected light flux cannot be greater than the difference between incident light quantity and losses into the surrounding tissue. (**b**) PPG waveform interpretation in the context of the obtained mathematical transfer function. The gray area represents the information lost, resulting in the diminution of the initial signal baseline.

The resulting mathematical relation between the mechanical part (deformation) and the optical one (absorption and reflection) is, in fact, a transfer function, as shown in Figure 6a. An input stimulus depicted as a black-colored waveform is applied to the obtained function. As a result, two output signals for vasoconstriction and vasodilation scenarios are shown. The greater the erythrocyte population in the illuminated volume site, the steeper the function slope, acting like an amplification effect. Conversely, in a lower cell population, the function is acting as an attenuator. The investigation presented in [52,53], between PPG waveform amplitude and vasomotor activity, agreed with this effect. In this way, the mathematical equation shows that the pulsatile origin of PPG is mainly linked to flow variation, which leads to the deformation of erythrocytes in the capillaries. In Figure 6b, the significance of the DC and AC parts of the optical signal is shown. As a result of Equation (2), a part of the incident light flux is lost by the transmissive effect, which is illustrated with a gray area. In reality, even if the collagen and elastin fibers pose the scattering property, the network still absorbs some degree of irradiant energy.

If the erythrocytes are motionless, the absorption value will increase exclusively with the cell’s concentration, total illuminated area, and average cosine angle, within the illuminated tissue volume. By slowly increasing the flow velocity, the absorption will increase too. At the diastolic phase, when the arterial pressure is at a minimum level, the imposed flow through the capillary network is low, leading to a diminished velocity gradient, *v_MIN_*. At the systolic phase, when the arterial pressure reaches the maximum value, the imposed flux rate through the peripheral network will increase accordingly, leading to a maximum velocity *v_MAX_*. As an effect, erythrocyte deformation will reach the maximum elongation, and, in turn, greater absorption phenomena will occur. The resulting PPG amplitude value at the maximum systolic point will be, in fact, the minimum.

Another outcome revealed by the obtained equation is that the higher the velocity baseline, the lower the recorded pulsatile amplitude will be. This effect results from the logarithmic law, where the partially derived reflection equation obtained by the velocity parameter leads to an inverse rational trend, 1/x. Therefore, a rapid flow of RBCs through the capillaries by taking a fixed transfer slope will result in a reduced pulsatile component during each cardiac cycle compared to a diminished flow rate scenario. But, in reality, the continuous physiological mechanism leads to a variable vasomotor state, which is equivalent to a variable transfer function. As a result, the same input stimuli can give, as output, a greater or diminished PPG amplitude. Therefore, the intuitive correlation given by Equation (5) between the PPG waveform and the blood pressure, represented by the imposed capillary flow rate, is misleading. The blood pressure assessment based on the absolute values of the optical signal shall be avoided [54,55,56].

The obtained mathematical model gives further insight into the pulse oximeter signal: motion artifact sources. If a slipping movement is considered, the illuminated volume boundary is changed, leading to a different number of irradiated red blood cells. A movement that disturbs the tissue arrangement, triggered by the muscle activity, will change the capillaries’ orientation to the incident flux. In other words, the initial fixed point on the transfer function will move within the available domain; thus, different signal components, the AC and DC part, will result in the same input stimulus.

## 4. Conclusions

In the microcirculation regime, two concomitant biological mechanisms took place: variable flow rate into the capillary network and red blood cell shape deformation. Every cardiac cycle modulates blood velocity through arterial trees; thus, RBCs are deformed accordingly. The main constituent of erythrocyte cells is the hemoglobin protein, which possesses optical properties, especially within the visible light spectrum. According to radiometric laws, an element absorbs light energy proportionate to the area exposed to the incident beam and the associated incident angle. Therefore, RBCs absorb light beams in a variable manner during the mechanical deformation process.

In the context of the photoplethysmography technique, the recorded optical signal embeds the aforementioned biomechanism. The resulting transfer function between the two physical domains elucidates the possible origin of the PPG baseline and pulsatile components. Firstly, the physical presence of the erythrocyte concentration within the illuminated volume contributes to the DC rate. The pulsatile nature of the waveform is related to the RBC variable paraboloid deformation during each imposed blood flow rate by the cardiac cycle. The logarithmic law that governs this mechanism contains a deformation saturation at higher flow velocities.

The obtained transfer function highlights the vasomotor activity impact in the resulting optical waveform. The vasodilation state that increases blood supply into the capillary network increases erythrocyte concentration too. Hence, the resulting transfer function acts like an amplification mechanism between the mechanical and optical quantities. The opposite effect, vasoconstriction, reduces the slope of the function, acting like an attenuator. This correlation shall not be associated with blood pressure values since different combinations of states between vasomotor activity and RBC velocity could lead to the same numerical value. It is important to note that the precapillary sphincter controls blood flow into the capillaries separately in the microcirculation case in order to maintain an efficient nutrient exchange.

These resulting findings from the proposed mathematical function are valuable for the development of the next sensor generation, especially on the wearable devices side. Information about RBC optical and mechanical behavior within the capillary network provides the needed engineering inputs for the PPG system design. Sensor parameters, such as the used light wavelength, optical power of the incident light flux, and beam orientation, with respect to the skin surface, could improve the quality of the recorded signal. Also, understanding the transfer function during the different physiological states will enhance the interpretation of the recorded waveform and will generate newer mitigation measures for the signal artifacts. All these changes in sensor system design would lead to novel hemodynamic parameters in order to improve healthcare monitoring. Wearable devices could prioritize this advantage over the existing ones, portability and enhanced computational power, to extend personalized healthcare availability.

At this point, the obtained PPG model, supported by the proposed mathematical transfer functions, points to a suitable microcirculation interpretation with respect to human physiology. Further work aims to conduct advanced investigations on the next arterial system level, macrocirculation, in order to be interfaced with the microcirculation side.

## Data Availability

All experimental data are available upon reasonable request from the corresponding authors via email.
